# Antigen-specific IgG glycosylation profiles in hamsters and macaques following COVID-19 vaccination

**DOI:** 10.1016/j.isci.2026.115400

**Published:** 2026-03-17

**Authors:** Bart Claushuis, Jan Nouta, Wenjun Wang, Carolien A.M. Koeleman, Arnoud H. de Ru, Peter A. van Veelen, Roland Zahn, Ramon Roozendaal, Gestur Vidarsson, Manfred Wuhrer

**Affiliations:** 1 Center for Proteomics and Metabolomics, Leiden University Medical Center, Leiden, the Netherlands; 2 Janssen Vaccines & Prevention B.V., Leiden, the Netherlands; 3 ZonMW, The Hague, the Netherlands; 4 Immunoglobulin Research Laboratory, Sanquin Research, Amsterdam, the Netherlands; 5 Department of Biomolecular Mass Spectrometry and Proteomics, Utrecht University, Utrecht, the Netherlands

**Keywords:** biological sciences, immunology, microbiology

## Abstract

Vaccination triggers the production of antigen-specific antibodies, including IgG. IgG molecules are glycosylated at the Fc region, and these glycan modifications markedly influence Fc receptor binding and downstream immune functions. Notably, infections with enveloped viruses such as SARS-CoV-2 can trigger the production of afucosylated IgG, which enhances FcγRIIIa binding and promotes antibody-dependent cellular cytotoxicity. Despite its importance, the glycosylation profiles of antigen-specific IgG following vaccination remain understudied, particularly in animal models. In this study, we investigated the Fc glycosylation patterns of antigen-specific IgG in hamsters and rhesus macaques following immunization with Ad26.COV2.S COVID-19 vaccine. Overall, our findings demonstrate that IgG Fc glycosylation dynamics in these animal models largely parallel those in humans. For example, we observed a transient afucosylated IgG response in both species, resembling the response previously reported in humans. These results indicate that IgG Fc glycosylation responses to vaccination in macaques and hamsters recapitulate key features of the human response, supporting their use as translational models for adenovirus vector-based vaccination studies.

## Highlights

• IgG Fc glycosylation patterns in hamsters and macaques upon Ad26.COV2.S vaccination
• A transient afucosylated IgG response is observed in hamsters and macaques
• Animal models recapitulate IgG glycosylation patterns observed in humans
• Similarities in glycosylation support the translational relevance of the animal models

## Graphical abstract

**Figure:**
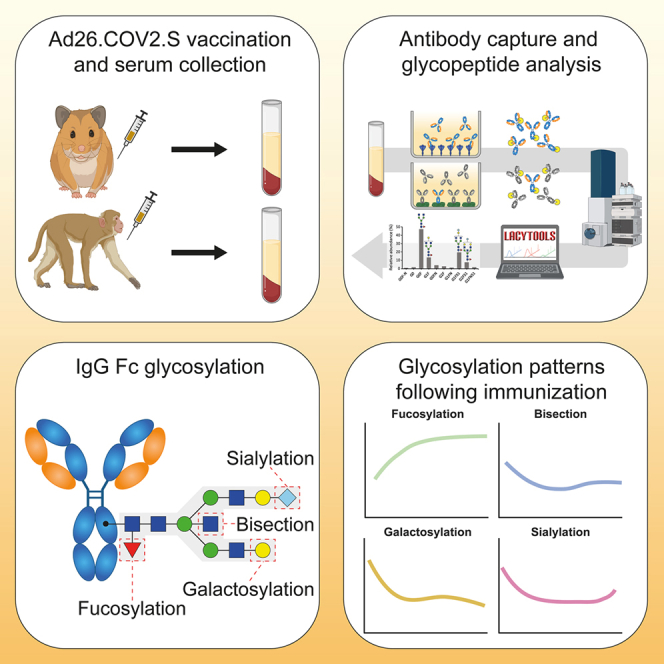

## Introduction

The global success of COVID-19 vaccines has highlighted the importance of robust humoral immunity in conferring protection against SARS-CoV-2. Antibody titers, particularly those targeting the viral Spike protein, have been widely adopted as correlates of vaccine-induced protection and are used to guide both regulatory approval and clinical monitoring.^1^^,^^2^ However, growing evidence suggests that antibody quality and, more specifically, Fc glycosylation, impact immune function following vaccination.^3^^,^^4^^,^^5^^,^^6^^,^^7^^,^^8^ For example, afucosylation of anti-dengue virus (DENV) IgG is associated with increased susceptibility to DENV infection postvaccination.^9^ Despite this, the glycosylation profiles of antigen-specific IgG following vaccination remain understudied, mostly due to technical challenges^10^^,^^11^ and particularly in the animal models that underpin preclinical vaccine development.^12^^,^^13^

**Figure 1:**
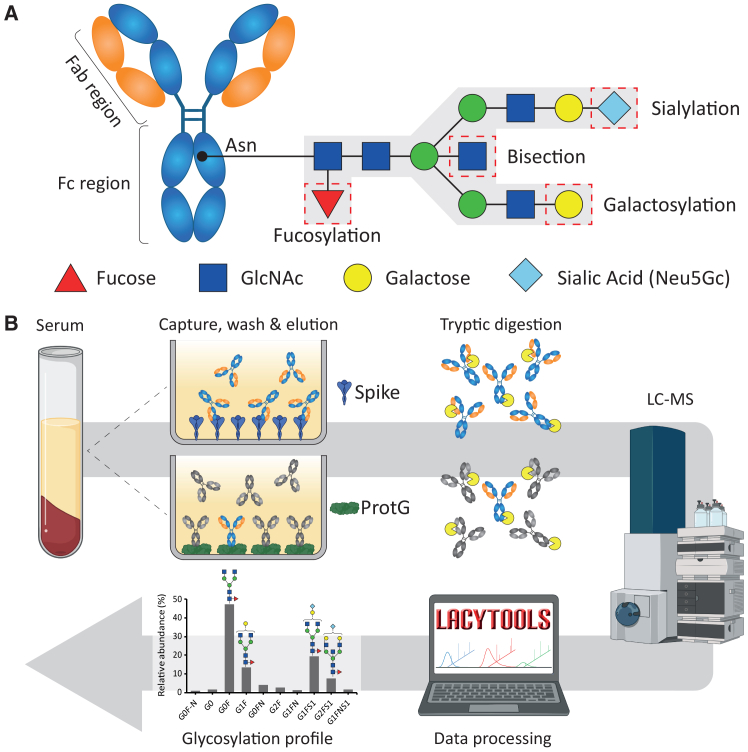
Analysis of IgG Fc glycopeptides (A) Schematic representation of IgG and the possible sugar modifications that are added to the core pentasaccharide. (B) Overview of the method used to analyze total and antigen-specific IgG.

IgG antibodies consist of two domains: the Fab region, which binds antigens and mediates neutralization, and the Fc region, which engages Fc gamma receptors (FcγRs) and complement to trigger effector functions such as antibody-dependent cellular cytotoxicity (ADCC), phagocytosis, and cytokine release.^14^ The Fc region contains a conserved *N*-linked glycan at asparagine 297, and its core pentasaccharide—which can be modified with a core fucose, bisecting *N*-acetylglucosamine (GlcNAc), galactoses, and sialic acids—modulates receptor affinity of the antibody (Figure 1A).^15^^,^^16^

These glycan modifications have well-characterized effects on IgG function. Afucosylated IgG displays up to 40-fold enhanced binding to FcγRIIIa and is associated with increased ADCC, inflammation, and disease severity in COVID-19 and other viral infections.^7^^,^^17^^,^^18^^,^^19^^,^^20^ Bisecting GlcNAc appears to have no effect on FcγR or C1q binding but may enhance ADCC indirectly by inhibiting core fucosylation.^21^^,^^22^ Galactosylation promotes IgG hexamerization and classical complement activation via C1q.^21^^,^^23^^,^^24^^,^^25^^,^^26^^,^^27^ Sialylation of the IgG Fc region has been proposed to attenuate inflammation,^28^^,^^29^^,^^30^ but recent evidence indicates that the effect of sialylation on FcγR-binding and activation of complement to be very modest, or not present.^21^^,^^24^^,^^25^^,^^31^ Together, these modifications tune IgG effector activity, making Fc glycosylation a central determinant of antibody-mediated immunity.

Recent studies indicate that the context of antigen presentation—specifically, whether the antigen is expressed on host cell membranes—can strongly influence Fc glycosylation.^8^ Enveloped viruses such as cytomegalovirus (CMV), HIV, and hepatitis B virus, which present viral glycoproteins on infected cell membranes, elicit highly afucosylated IgG responses.^7^ Alloimmune responses against membrane-bound host antigens (e.g., in fetal and neonatal alloimmune thrombocytopenia) similarly induce afucosylated antibodies.^7^^,^^32^^,^^33^ Notably, vaccines that lead to membrane-associated antigen expression, i.e., mRNA, viral vector, and live attenuated vaccines of enveloped viruses, produce a similar afucosylated IgG response, whereas subunit and inactivated virus vaccines do not.^7^^,^^34^

SARS-CoV-2 is an enveloped virus that assembles intracellularly in the endoplasmic reticulum-golgi intermediate compartment (ERGIC), rather than at the plasma membrane.^35^^,^^36^ However, due to trafficking sequences in the cytoplasmic tail, a fraction of the spike protein localizes to the cell surface, where it contributes to cell-cell fusion (syncytia) and becomes immunologically visible.^37^^,^^38^^,^^39^^,^^40^ This type of membrane presentation may, among other factors, explain the intermediate levels of afucosylation observed in anti-Spike protein (anti-S) IgG, which are lower than responses to viruses such as CMV but still detectable in infection and following vaccination.^6^^,^^7^^,^^17^^,^^41^

Although Fc glycosylation clearly modulates immune function, data on antigen-specific IgG glycosylation in animal models are extremely scarce, and the degree to which these profiles mirror human responses in the context of disease and vaccination is unclear. IgG glycan structures are broadly conserved across species, but their glycosylation profiles often differ. For example, rhesus macaques (*Macaca mulatta*) exhibit higher baseline bisection, galactosylation, and sialylation, and incorporate the sialic acid *N*-glycolylneuraminic acid (Neu5Gc), which humans lack.^42^^,^^43^^,^^44^ Although some differences exist in IgG subclasses and Fcγ receptors,^45^^,^^46^^,^^47^^,^^48^^,^^49^ rhesus macaques are valuable models due to their otherwise genomic and immunological similarity to humans,^42^^,^^50^^,^^51^ e.g., the increased binding of afucosylated IgG to FcγRIIIa.^51^

Syrian golden hamsters (*Mesocricetus auratus*) are widely used for SARS-CoV-2 research due to their natural susceptibility to the virus, in contrast to other rodents such as mice, and their recapitulation of key pathological and transmission features of COVID-19.^52^^,^^53^ Yet, their IgG Fc glycosylation patterns remain hitherto poorly defined. Furthermore, unlike in humans and macaques, the functional implications of IgG Fc glycosylation have not yet been investigated in hamsters.

In this study, we analyzed antigen-specific IgG Fc glycosylation in Syrian golden hamsters and rhesus macaques following SARS-CoV-2 vaccination and compared these profiles to human data. By examining subclass distribution and glycan composition, we assess the extent to which these animal models reflect human Fc glycosylation dynamics in COVID-19 vaccination and evaluate their utility in preclinical vaccine research. This work addresses a critical gap in translational immunology and may inform the design and interpretation of future vaccine studies.

## Results and discussion

### IgG subclass identification and glycopeptide profiling in hamsters and macaques

**Figure 2:**
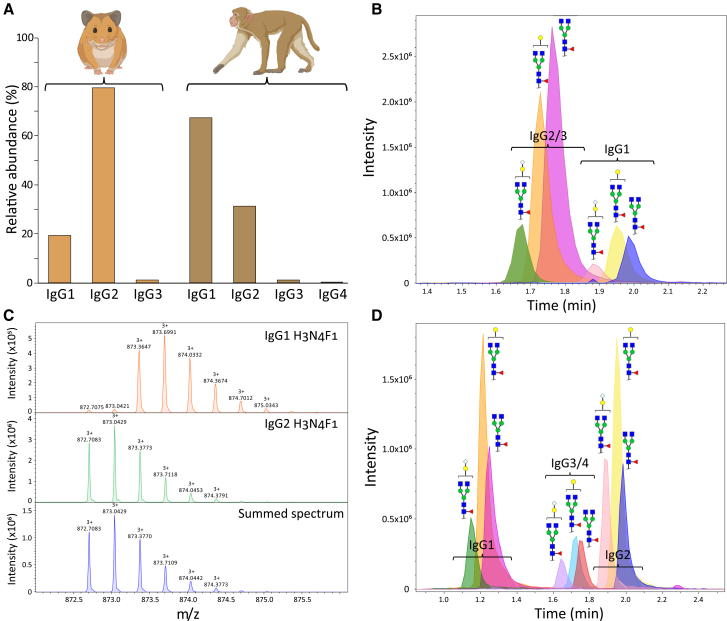
IgG subclasses in Syrian Golden Hamsters and Rhesus Macaques (A) Relative abundances of the IgG subclasses determined by the LC-MS/MS analysis of purified IgG from a commercially available hamster serum and pooled serum from seven healthy macaques. (B) Hamster IgG Fc glycopeptide clusters observed after total IgG capture. Extracted ion chromatograms of three glycopeptide species per subclass are shown. The glycan compositions are indicated as follows: green circle = mannose; yellow circle = galactose; blue square = N-acetylglucosamine; red triangle = fucose; light-blue diamond = N-glycolylneuraminic (sialic) acid. Glycopeptide species were assigned based on accurate mass. (C) Isotopic patterns of hamster IgG1 and IgG2 H3N4F1 glycopeptides and a spectrum depicting the summed intensities of both molecules. The glycan compositions are indicated as follows: H = hexose; N = N-acetylhexosamine; F = deoxyhexose (fucose). (D) Macaque IgG Fc glycopeptide clusters observed after total IgG capture. Extracted ion chromatograms of three glycopeptide species are indicated. The glycan compositions are as in (B). Glycopeptide species were assigned based on accurate mass.

To characterize IgG subclass distribution in Syrian golden hamsters (*Mesocricetus auratus*, NCBI: txid10036) and rhesus macaques (*Macaca mulatta*, NCBI: txid9544), we performed LC-MS/MS analysis of total IgG. Using the GLYcoLISA method (Figure 1B;^11^) coupled with protein G capture, we purified total IgG from a commercially available hamster serum and pooled serum from seven healthy macaques.^42^^,^^54^ In hamsters, three IgG subclasses are annotated in the NCBI protein database (Table S1). Liquid chromatography with tandem mass spectrometry (LC-MS/MS) revealed the presence of all three, with IgG2 as the most abundant (Figure 2A). Similarly, all four known macaque IgG subclasses were detected, with IgG1 as the predominant subclass (Figure 2A).^45^^,^^55^

Next, we profiled Fc glycosylation in each species. In hamsters, IgG2 and IgG3 possess identical tryptic Fc glycopeptides (Table 1), preventing their separation by LC-MS (Figure 2B). Given the dominance of IgG2, glycopeptide signals were attributed primarily to this subclass. IgG1 and IgG2/3 tryptic Fc glycopeptides differ by only ∼2 Da (Table 1), complicating their resolution due to an isotopic overlap in summed spectra (Figure 2C) that precludes accurate quantification with our processing method.^56^ Consequently, subsequent hamster analyses focused on IgG2 glycopeptides.

**Table 1:** IgG subclasses and glycopeptides of Syrian Golden hamsters (*Mesocricetus auratus*) and Rhesus macaques (*Macaca mulatta*)

Organism	IgG subclass	Tryptic Fc glycopeptide	Mass (monoisotopic)
Syrian Golden hamster	IgG1	EEQF**N**STYR	1173.517
Syrian Golden hamster	IgG2	QQQF**N**STYR	1171.549
Syrian Golden hamster	IgG3	QQQF**N**STYR	1171.549
Rhesus macaque	IgG1	ETQY**N**STYR	1161.517
Rhesus macaque	IgG2	EEQF**N**STYR	1173.517
Rhesus macaque	IgG3	QF**N**STYR	915.432
Rhesus macaque	IgG4	QF**N**STYR	915.432

The asparagine residue containing the *N*-glycan is shown in bold.

In macaques, the IgG3 and IgG4 glycopeptides are identical, precluding their separation by LC-MS (Figure 2D). However, as these subclasses constitute only a minor part of the total IgG, we decided to focus on the most abundant subclass, IgG1, for glycan analysis.

In total, we identified 12 IgG2 glycopeptides in the commercially available hamster serum (Figure S1A) and 14 IgG1 glycopeptides in the macaque serum (Figure S1B). The identity of several glycopeptides was confirmed by LC-MS/MS (Figure S2).

### Glycosylation of IgG2 following vaccination with Ad26.COV2.S in Syrian golden hamsters

Syrian golden hamsters recapitulate many aspects of SARS-CoV-2 infection that laboratory mice do not. Hamster ACE2 binds the SARS-CoV-2 Spike protein with high affinity, allowing productive infection, whereas mouse ACE2 has poor affinity for Spike, rendering wild-type mice largely insusceptible to infection.^57^^,^^58^ Only by using mouse-adapted viral strains or transgenic mice expressing human ACE2 can productive infection of SARS-CoV-2 be achieved, approaches which can introduce confounding adaptations to either the virus or the organs/cells expressing ACE2. Hamsters, by contrast, develop respiratory disease, pathology, and transmission dynamics similar to mild human COVID-19, making them a convenient and relevant small animal model for vaccine studies.

**Figure 3:**
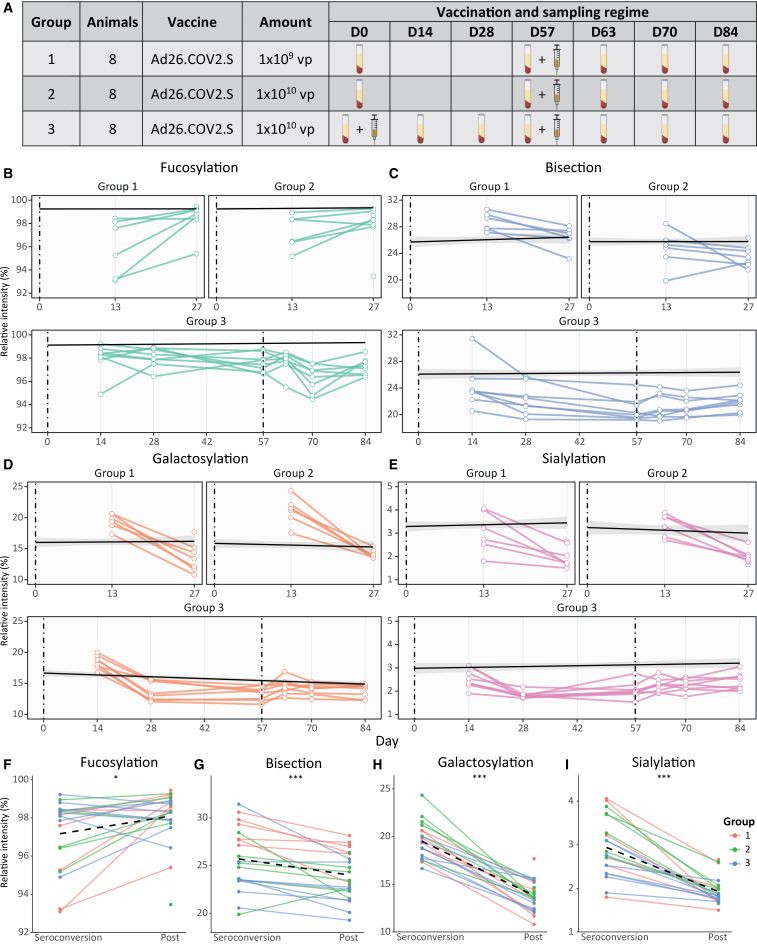
IgG2 Fc glycosylation traits in hamsters (A) The vaccination and sampling regime for three groups of female hamsters. (B–E) The relative intensities of anti-S IgG2 Fc (B) fucosylation, (C) bisection, (D) galactosylation, and (E) sialylation for each group of animals. The mean total IgG and the standard error (SE) are depicted by the solid black line and the gray ribbon, respectively. The dashed vertical lines indicate the moment of vaccination. The moment of the first vaccination is set to day 0. (F–I) Comparison of the relative intensities of the derived traits (F) fucosylation, (G) bisection, (H) galactosylation, (I) and sialylation at seroconversion and post (2 weeks after) compared by Wilcoxon signed-rank test. The dashed line represents the change in the mean for each glycosylation trait. ∗*p* < 0.05, ∗∗*p* < 0.01, and ∗∗∗*p* < 0.001.

To assess vaccination-induced changes in glycosylation, three groups of female hamsters were immunized with varying doses of the Ad26.COV2.S vaccine and serum were collected longitudinally (Figure 3A). Group 1 received 1×10^9^ viral particles (vp), group 2 received 1×10^10^ vp, and group 3 received two doses of 1×10^10^ vp (days 0 and 57). All serum samples were processed using our standard method for antibody glycoprofiling (Figure 1B), and antibodies were purified using protein G and Spike protein-coated wells to capture total IgG and anti-S IgG, respectively.

We examined the four Fc glycosylation traits: fucosylation, bisection, galactosylation, and sialylation (Figure 1A). The definitions and calculations for each trait are shown in Table S2. Ten IgG2 glycopeptides, selected based on their presence in anti-S samples, were included in the analyses (Table S3).

Total IgG2 remained constant over time, while anti-S IgG2 became detectable ∼2 weeks post-vaccination and increased thereafter (Figure S3A). Total IgG2 was heavily fucosylated, with around 99% of the glycans carrying a core fucose, whereas anti-S IgG2 displayed lower fucosylation (Figures 3B and S3B). In groups 1 and 2, anti-S IgG2 displayed lower fucosylation (∼93–99%, median ∼97%) at seroconversion, consistent with the transient afucosylated response observed in humans for membrane-embedded antigens.^7^ For group 3, lower fucosylation levels after the first dose are less evident (Figure 3B). Still, anti-S IgG2 fucosylation was statistically significantly lower compared to the fucosylation levels 2 weeks after seroconversion (post) across all groups (Figure 3F).

The hamsters in group 3 received a second dose of the Ad26.COV2.S vaccine, which significantly reduced fucosylation of anti-S IgG2 13 days later (day 70; Figures 3B and S4A). Collectively, seroconversion by vaccination with Ad26.COV2.S elicits a transient afucosylated response in hamsters, as is observed in humans.^41^ However, apart from a small subset of individuals, IgG afucosylation after a second dose is not observed in humans with the Ad26.COV2.S vaccine or other COVID-19 vaccines, possibly indicating species-specific regulatory mechanisms.^6^^,^^41^ Although the differences in fucosylation levels appear relatively small at first glance, the relative increase between, e.g., 1% afucosylated glycopeptides and 3% afucosylated glycopeptides is 200%, which might significantly affect the immune response in a context of natural infection by promoting ADCC and inflammation.^7^^,^^17^^,^^20^

In all three groups, bisection of anti-S IgG2 was highest at seroconversion (∼2 weeks post-vaccination) and declined thereafter (Figures 3C–3G). In line with this, we observed a negative correlation between the levels of bisection and the amount of IgG2 (Figure S3C), i.e., bisection is highest at lower levels of IgG2. In group 3 (two-dose regimen), bisection decreased after the first dose until the administration of the second dose, which elevated bisection levels until the end of the study (Figures 3C and S4B). Overall, both total and anti-S IgG2 bisection levels were higher in hamsters (∼25%) than in human cohorts (∼10–15% total, ∼2.5–10% anti-S).^6^^,^^7^^,^^34^^,^^41^ Interestingly, in humans, bisection of IgG increases over time following vaccination with COVID-19 vaccines,^41^ while in hamsters, a decrease is observed, again indicating a species-specific regulation of IgG glycosylation.

The dynamics of galactosylation and sialylation levels in hamsters were highly similar, reflecting the fact that sialylation requires a terminal galactose. For anti-S IgG2, both glycosylation traits were highest at seroconversion and decreased afterward in all three groups (Figures 3D, 3E, 3H, and 3I). In addition, a negative correlation was observed between the amount of galactosylation and IgG2 levels (Figure S3D). A similar negative correlation was not observed for sialylation (Figure S3E). These dynamics align with human responses to Ad26.COV2.S but differ from other COVID-19 vaccines.^41^ Although similar dynamics are observed in hamsters and humans, the observed levels of hamster anti-S galactosylation (∼17–24%) and sialylation (∼2–4%) were much lower compared to those in humans (∼68–89% and ∼12–23%, respectively) two weeks post-vaccination.^41^ After the administration of the second dose in group 3, both traits increased again, mirroring the initial response. However, in contrast to the other glycosylation traits, the second dose resulted in a quick but short-lived response with galactosylation levels significantly rising within one week (Figure S4C), while sialylation levels increased more gradually (Figure S4D).

The relatively high levels of galactosylation at seroconversion may indicate increased IgG hexamerization capacity, as IgG galactosylation promotes hexamer formation and C1q binding for complement activation.^21^^,^^23^^,^^24^^,^^26^^,^^27^ Hence, the increase in galactosylation early after immunization suggests higher complement activation potential. After the second dose in group 3, both galactosylation and sialylation rose again (Figures 3D, 3E, S4C, and S4D), an effect also observed in humans receiving COVID-19 booster immunizations.^6^^,^^41^

Overall, the early glycosylation patterns observed in hamsters following vaccination closely resemble those seen in humans, with the notable exception of bisection, which exhibits an opposite trend. The similarities in glycosylation trends between hamsters and humans support the translational relevance of the hamster model. This underlines its suitability as a representative rodent system for investigating vaccine efficacy and the pathophysiological impact of infectious agents such as SARS-CoV-2.

### Glycosylation of IgG1 following vaccination with Ad26.COV2.S in rhesus macaques

**Figure 4:**
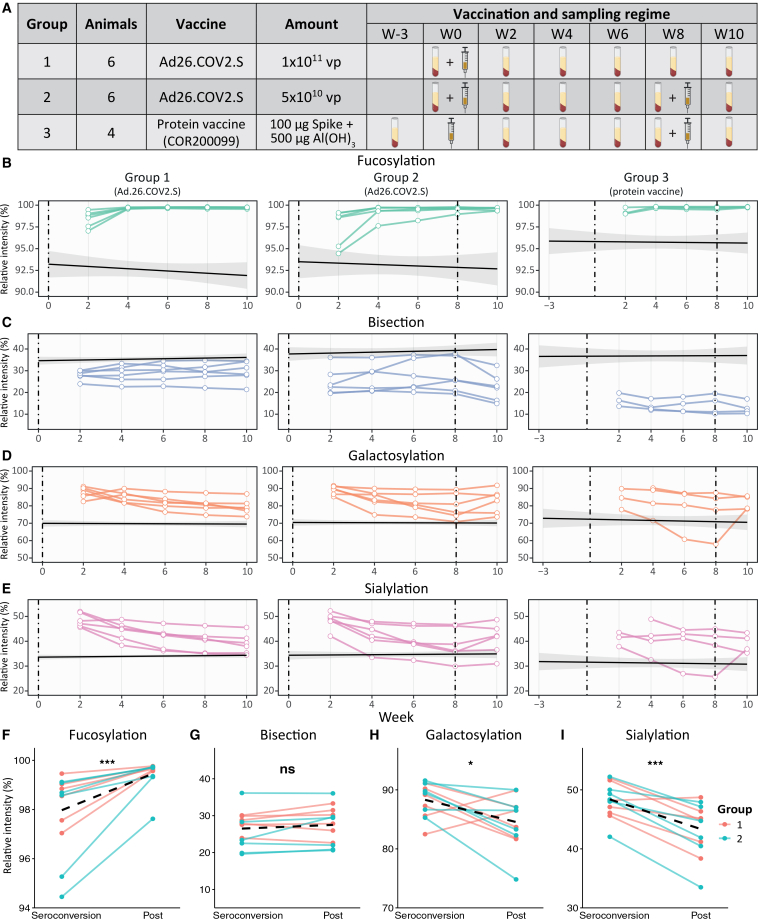
IgG1 Fc glycosylation traits in macaques (A) The vaccination and sampling regime for three groups of female macaques. (B–E) The relative intensities of anti-S IgG1 Fc (B) fucosylation, (C) bisection, (D) galactosylation, and (E) sialylation for each group of animals. The mean total IgG and the SE are depicted by the solid black line and the gray ribbon, respectively. The dashed vertical lines indicate the moment of vaccination. The moment of the first vaccination is set to day 0. (F–I) Comparison of the relative intensities of the derived traits (F) fucosylation, (G) bisection, (H) galactosylation, (I) and sialylation at seroconversion and post (2 weeks after) for groups 1 and 2, compared by the Wilcoxon signed-rank test. The dashed line represents the change in the mean for each glycosylation trait. ∗*p* < 0.05, ∗∗*p* < 0.01, and ∗∗∗*p* < 0.001.

To investigate IgG Fc glycosylation in an animal model more closely resembling humans, we examined female macaques vaccinated with either Ad26.COV2.S or a protein-based Spike vaccine (Figure 4A). Group 1 received a single dose of 1×10^11^ vp; group 2 received two doses of 5×10^10^ vp (weeks 0 and 8); and group 3 received the protein vaccine following the same schedule.^59^ In contrast to the viral vector vaccine Ad26.COV2.S, the protein vaccine, will not result in the presentation of the Spike protein on the host’s cell membrane. Based on the observations in humans in various immunization settings,^7^ we would not expect an afucosylated IgG response after vaccination with the protein vaccine. Sample processing and data analysis proceeded as for the hamsters. The description and calculation of glycosylation traits are shown in Table S2. The 13 IgG1 glycopeptides that were included in the analyses were selected based on their presence in anti-S samples and are shown in Table S4.

Total IgG levels were found to be stable throughout the study period (Figure S5A). Total IgG Fc fucosylation levels closely resemble those in humans. In the human population, approximately 94% of total IgG is fucosylated.^41^ In macaques, the median fucosylation level of total IgG1 is similar, at 94.3%, indicating a comparable baseline fucosylation pattern between the two species.

In contrast, anti-S IgG1, first observed two weeks post-vaccination (Figures 4 and S5A), showed higher fucosylation levels (median 99.7%) compared to total IgG1 (Figures 4B and S5B). Groups 1 and 2 (Ad26.COV2.S vaccine), which received a dose at week 0, demonstrated a modest but consistent lower fucosylation of anti-S IgG1 at seroconversion (week 2), ranging from 94.5% to 99.5% (Figure 4B). Due to limited sample sizes and only a 2-fold difference in vp dose between groups 1 and 2, these groups were pooled for statistical analysis. This revealed a significantly lower fucosylation at seroconversion (week 2) compared to post (week 4) (Figure 4F), indicating a transient afucosylated IgG1 response early after immunization, as was seen in both hamsters (Figure 3B) and humans.^41^ Furthermore, a positive correlation was observed between the IgG intensities and the level of fucosylation (Figure S5B).

Group 2 (Ad26.COV2.S vaccine) animals received a second vaccine dose, yet no further decrease in fucosylation was observed two weeks post-vaccination (Figures 4B and S6). This pattern is consistent with observations in human COVID-19 vaccination studies, in which only a small subset of individuals showed an afucosylated response following a booster vaccine.^6^^,^^41^

Group 3 animals, which received a protein-based vaccine not expected to elicit an afucosylated response due to the absence of membrane-embedded antigens, showed minimal changes (Figure 4B). Two out of four animals exhibited slightly reduced fucosylation at early time points (99.0% and 99.1%) relative to later sampling. In one animal, anti-S IgG1 was detectable only from week 4 onwards. Following the second dose at week 8, no appreciable changes in fucosylation levels were observed in group 3 (Figures 4B and S6). However, due to the small cohort size, statistical analysis was not meaningful, underscoring the need for larger sample sizes to robustly assess the impact of protein vaccination on IgG1 fucosylation.

In human vaccinees who received the Ad26.COV2.S vaccine, bisection is lowest at seroconversion and increases slightly over time.^41^ This upward trend in bisection is seen in several animals of groups 1 and 2 (Ad26.COV2.S vaccine), but no significant increase is observed on the whole (Figures 4C–4G). In line with this, and in contrast to the hamsters, no correlation is observed between the levels of IgG1 and bisection (Figure S5C). Both the total (median 32.3%) and anti-S (median 23.7%) IgG1 bisection levels are higher in macaques compared to humans (∼10–15% and ∼2.5–10%, respectively). However, animals in group 2 that were administered a second dose showed a significant reduction in bisection 2 weeks post-vaccination (Figures 4C and S6), as was observed in human vaccinees receiving a booster.^41^

Galactosylation and sialylation dynamics after vaccination in macaques are very similar to the dynamics in hamsters (Figures 3D and 3E) and humans.^41^ Both glycosylation traits are highest at seroconversion and decrease afterward (Figures 4D, 4E, 4H, and 4I), and their levels are negatively correlated with IgG1 intensities (Figures S5D and S5E). The levels of galactosylation are comparable to those in humans, but the levels of sialylation in macaques are ∼3-fold higher than those in humans.^41^ In both hamsters and humans, a second dose increases galactosylation and sialylation (Figure S4;^41^). In the macaques from group 1 (single-dose regimen), these traits remain declining after week 8 (Figures 4D, 4E, and S6). For the macaques in group 2, galactosylation and sialylation increased after the second dose (week 8) in most animals, indicating an effect of the booster compared to group 1, although this increase was not statistically significant (Figures 4D, 4E, and S6). For group 3 (protein vaccine), a steep increase in these traits was observed for only a single animal, but similar changes are absent in the other animals that received a second dose of the protein vaccine.

**Figure 5:**
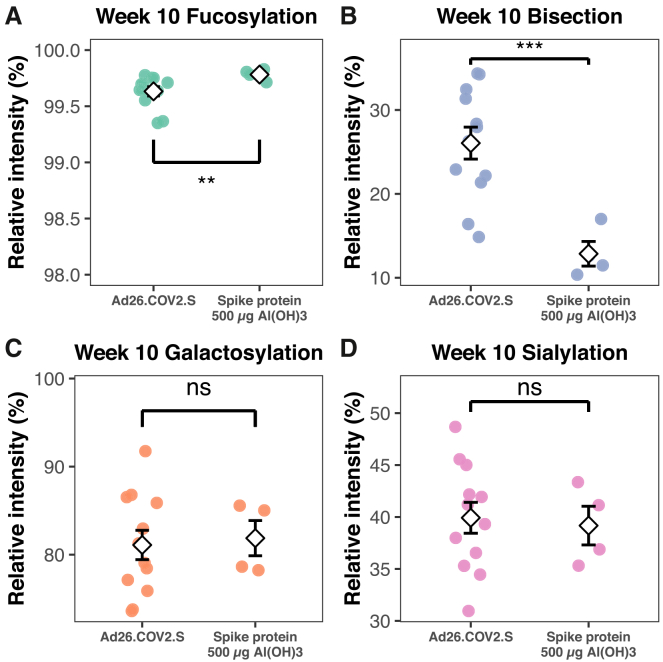
Effect of vaccine type on anti-S IgG1 Fc glycosylation in macaques at week 10 Levels of (A) fucosylation, (B) bisection, (C) galactosylation, and (D) sialylation at week 10 for macaques vaccinated with either Ad26.COV2.S or the Spike protein vaccine. The white diamonds show the group means, and the error bars represent the SE. Differences between groups were assessed by the Mann-Whitney U test (A) and the Welch’s *t* test (B–D). ∗*p* < 0.05, ∗∗*p* < 0.01, and ∗∗∗*p* < 0.001.

To investigate whether the vaccine type influenced IgG1 Fc glycosylation, we compared glycosylation traits levels at the study’s final time point, i.e., week 10 (Figure 5). Although both the sample sizes and the absolute differences were small, IgG1 fucosylation was slightly lower in the macaques that received the Ad26.COV2.S vaccine compared to those that received the Spike protein vaccine at week 10 (Figure 5A). There is a clearer distinction between the bisection levels of the two different vaccine types at week 10 (Figure 5B). The macaques used in this study were vaccinated with either Ad26.COV2.S or the Spike protein vaccine was previously shown to differ in the production of cytokines by CD4^+^ T cells at week 10.^59^ The Ad26.COV2.S vaccine induces the production of mainly Th1 cytokines, while the protein vaccine shows a more dominant Th2 response.^59^ Several cytokines, including IFN-γ, have been shown to promote the bisection of IgG,^60^ and IFN-γ was elevated in the macaques receiving the Ad26.COV2.S vaccine at week 10.^59^ Possibly, the increased levels of IFN-γ—among other differences in cytokines—explain the higher bisection levels in groups 1 and 2 at week 10. For the galactosylation and sialylation traits, no differences between vaccine types were observed (Figures 5C and 5D).

Compared to hamsters, rhesus macaques are evolutionarily closer to humans, and indeed their immune responses and IgG Fc domain biology tend to more closely approximate humans. This makes macaques extremely valuable for assessing vaccine responses and antibody functionality in a preclinical setting. However, non-human primate models come with practical and ethical constraints. They are expensive to house and manage and require specialized facilities. Accordingly, sample sizes are often limited—in our study, each group comprised only 4–6 macaques. Despite this, we were able to detect statistically significant changes in Fc glycosylation (e.g., the anti-S IgG1 afucosylation at week 2), underscoring that glycopeptide profiling using our method is a sensitive and robust approach.

### Concluding remarks and future perspectives

In this study, we profiled antigen-specific IgG Fc glycosylation at the glycopeptide level in animal models of vaccination. By directly comparing a small rodent and a macaque model, we demonstrate that both can recapitulate the glycosylation shifts seen in humans, such as transient afucosylation, and vaccine-induced increases in galactosylation and sialylation. However, differences were also observed, such as in the baseline glycosylation state of both total and antigen-specific IgG, as well as the bisection dynamics between hamsters and humans. These findings fill an important gap in translational immunology, as animal vaccine studies rarely examine IgG Fc glycosylation. While the qualitative effects of IgG Fc glycosylation on receptor binding—and thus on the immune response—are only partially characterized in macaques and remain undefined in hamsters, the similarities in glycosylation dynamics across hamsters, macaques, and humans suggest that these findings may be translatable to humans, where these effects are better understood.

Nonetheless, further research is needed to examine glycosylation dynamics in the context of infection, e.g., with both enveloped and non-enveloped viruses, as well as following immunization with various vaccines and vaccine platforms. In addition, incorporating clinical outcomes such as protection from infection or disease severity is a valuable variables that aid in assessing the biological relevance of IgG glycosylation traits, e.g., whether IgG Fc fucosylation levels correlate with protection from infectious agents. Although longitudinal shifts in fucosylation are modest in absolute terms (e.g., 95%–99%), the corresponding relative differences in afucosylated IgG can be substantial and may meaningfully affect effector functions; for instance, 5% afucosylation represents a 5-fold increase relative to 1%. Future studies should therefore aim to define the qualitative and functional consequences of these apparently small changes, e.g., the differences in disease severity in the context of infection. Moreover, since IgG Fc glycosylation is influenced by sex hormones,^61^^,^^62^ studies should include male subjects—as opposed to this study, which only included female subjects—to identify sex-specific IgG Fc glycosylation patterns.

Moving forward, it would benefit the scientific field to re-examine stored sera from past animal studies (e.g., preclinical trials of vaccines or infection models) to investigate IgG Fc glycosylation, rather than relying on new animal experiments. Such retrospective analyses can expand our knowledge while avoiding unnecessary animal use. In conclusion, while animal models remain indispensable for preclinical vaccine assessment, one must account for species-specific differences in IgG Fc glycosylation when translating these results to humans. Improved understanding of these differences will enable better modeling of vaccine-induced antibody quality and help in the design of vaccines that optimally engage the human immune system.

### Limitations of the study

This study has several limitations. First, sample sizes were modest: Hamster groups comprised eight animals each, and macaque groups comprised four to six animals. To achieve sufficient statistical power, groups were combined for certain analyses. For example, in hamsters, the comparison of Fc glycosylation traits at seroconversion and at two weeks post-seroconversion included eight animals vaccinated with 10^9^ vp and sixteen animals vaccinated with 10^10^ vp. While the longitudinal direction of shifts in glycosylation traits was consistent across dose groups, vaccine dose may influence the magnitude of these shifts. Despite these constraints, we nonetheless detected statistically significant changes in IgG Fc glycosylation over time.

Second, whereas the functional consequences of IgG Fc glycosylation have been studied in humans and macaques, comparable data are lacking for hamster IgG and hamster Fc receptors. Consequently, we cannot predict effector functions from the observed hamster glycosylation profiles. Nevertheless, the hamster Fc glycosylation patterns observed following Ad26.COV2.S vaccination largely resembled those in macaques and humans, supporting the translational utility of the hamster model for preclinical studies.

Third, the study did not include clinical endpoints (e.g., protection from infection or disease severity) following challenge. Incorporating such outcomes would strengthen the interpretation of the biological relevance of inter-animal or inter-group differences in IgG Fc glycosylation after vaccination.

## Resource availability

### Lead contact

Requests for further information and resources should be directed to and will be fulfilled by the lead contact, Bart Claushuis (b.claushuis@lumc.nl).

### Materials availability

This study did not generate new unique reagents.

### Data and code availability

• Data: The mass spectrometry proteomics data have been deposited to the ProteomeXchange Consortium via the PRIDE^63^ partner repository and are publicly available as of the date of publication. Accession numbers are listed in the key resources table.
• Code: This study did not create new code.
• Other items: No other resources have been created in this study.

## Acknowledgments

Not applicable.

## Author contributions

B.C.: formal analysis, visualization, writing – original draft, and writing – review and editing. J.N: formal analysis, investigation, methodology, visualization, writing – original draft, and writing – review and editing. W.W., C.A.M.K., and A.H.d.R.: investigationand writing – review and editing. P.A.v.V.: resources and writing – review and editing. R.Z.: resources and writing – review and editing. R.R.: conceptualization, investigation, methodology, resources, supervision, and writing – review and editing. G.V.: conceptualization and writing – review and editing. M.W.: conceptualization, supervision, and writing – review and editing.

## Declaration of interests

R.R. and R.Z. were employees of Janssen Vaccines & Prevention B.V., a Janssen Pharmaceutical Company of Johnson & Johnson, at the time of the study, and may have ownership of shares in Johnson & Johnson. R.Z. is a coinventor on provisional vaccine patents (62/969,008; 62/994,630). All other authors declare no competing interests.

## Declaration of generative AI and AI-assisted technologies in the writing process

During the preparation of this work, the author(s) used ChatGPT (GPT-4o and GPT-5) in order to improve readability and language. After using this tool/service, the author(s) reviewed and edited the content as needed and take(s) full responsibility for the content of the published article.

## STAR★Methods

### Key resources table

**Table:** 

REAGENT or RESOURCE	SOURCE	IDENTIFIER
**Biological samples**		

Syrian golden hamster serum	Abcam	Ab74834
Rhesus macaque serum	Khatri et al.^54^; Petralia et al.^42^	N/A

**Chemical, peptides, and recombinant proteins**		

Ad26.COV2.S	Bos et al.^64^	N/A
Spike protein full-length (COR200099)	Bos et al.^64^	N/A
Aluminum hydroxide adjuvant (2% alhydrogel)	Invivogen	Vac-alu-50
TPCK trypsin	Promega	V5111
Protein G Sepharose beads	GE healthcare	Cat# GE17-0618-05

**Deposited data**		

Raw LC-MS data	This paper	ProteomeXchange ID: PXD068257

**Experimental models: Organisms/strains**		

*Macaca mulatta*; Rhesus macaque (Indian origin)	Solforosi et al.^59^	N/A
*Mesocricetus auratus*; Syrian Golden Hamster	Janvier Labs	Rj:AURA

**Software and algorithms**		

Rstudio version 2024.12.1	Posit Software PBC	https://posit.co/download/rstudio-desktop/
R version 4.4.2	The R Project for Statistical Computing	https://www.r-project.org/
Proteome discoverer version 2.5.0.400	Thermo Fisher Scientific	https://www.thermofisher.com/nl/en/home/industrial/mass-spectrometry/liquid-chromatography-mass-spectrometry-lc-ms/lc-ms-software/multi-omics-data-analysis/proteome-discoverer-software.html?erpType=Global_E1
Mascot version 2.2.7	Matrix Science	www.matrixscience.com
Bruker Compass DataAnalysis v5.0	Bruker Daltonics	https://bruker-compass-dataanalysis.software.informer.com/
MSConvert (Proteowizard v3.0.8708)	Proteowizard	https://proteowizard.sourceforge.io/
LaCyTools v1.99	Jansen et al.^56^	N/A

**Other**		

Nunc immunoplate Maxisorp 96-well plate	Thermo Fisher Scientific	cat# 442404
Protein G Sepharose beads	GE healthcare	GE17-0618-01
Oro-Flex Filterplate 96-wells	Orochem	OF1100PE07
Dionex Ultimate3000nano HPLC	Thermo Fisher Scientific	N/A
Exploris480 mass spectrometer	Thermo Fisher Scientific	BRE725533
Impact HD Q-TOF mass spectrometer	Bruker Daltonics	N/A

### Experimental model and study participant details

All animal procedures were reviewed and approved by the Central Authority for Scientific Procedures on Animals (Centrale Commissie Dierproeven; CCD) and conducted under EU animal testing directive 2010/63/EU, ETS 123, and relevant Dutch regulations for animal experiments. The hamster and macaque studies were approved by the CCD under reference numbers AVD4010020209446 and AVD5020020209404, respectively.

The study using hamsters was performed at Wageningen Bioveterinary Research (WBVR), Lelystad, The Netherlands. Female Syrian golden hamsters (Rj:AURA), aged 9 to 14 weeks, were sourced from Janvier Labs (France). Group 1 (*n* = 8) received a single dose with 1 x 10^9^ vp of Ad26.COV2.S at day 57, and group 2 (*n* = 8) received 1 x 10^10^ vp of Ad26.COV2.S at day 57. Group 3 (*n* = 8) was immunized twice with 1 x 10^10^ vp of Ad26.COV2.S at day 0 and day 57. The hamsters received intramuscular vaccinations (100 μl) while under general anesthesia using either isoflurane or a combination of medetomidine and ketamine administered intraperitoneally. Anesthesia was reversed using subcutaneous atipamezole. Blood was collected from the retro-orbital sinus under anesthesia, following the schedule shown in Figure 3A. No influence of sex on the results could be reported, since only female hamsters were included in this study.

The study using macaques was performed at Biomedical Primate Research Centre, Rijswijk, Netherlands. Female rhesus macaques (*Macaca mulatta*) of Indian origin, aged 13.8 to 21.9 years and weighing between 6.6 and 12.6 kg, were assigned to four treatment groups and housed in ABSL-III conditions, pair-housed with compatible partners, as previously described.^59^ Before the start of the study, all animals were surgically implanted with an AnipillV2 telemetry device (BodyCAP) in the abdominal cavity to record body temperature every 15 minutes. Group 1 (*n* = 6) received a single dose of 10^11^ viral particles (vp) of Ad26.COV2.S at week 0. Group 2 (*n* = 6) was immunized with 5 × 10^10^ vp of Ad26.COV2.S at weeks 0 and 8. Group 3 (*n* = 4) received 100 μg of spike protein with 500 μg of aluminum hydroxide adjuvant (2% Alhydrogel; InvivoGen) at weeks 0 and 8. Group 4 (*n* = 4), serving as the sham control, received 10^11^ vp of Ad26.RSV.gLuc. As these animals did not generate anti-spike antibodies, their data were excluded from the Results section. All immunizations were administered intramuscularly into the quadriceps of the left hind leg. Blood samples were collected following the schedule shown in Figure 4A. No influence of sex on the results could be reported, since only female macaques were included in this study.

### Method details

#### Vaccines

The development of the Ad26.COV2.S vaccine was previously described.^64^ In summary, it consists of a non-replicating Ad26 viral vector carrying a gene encoding a prefusion-stabilized SARS-CoV-2 spike protein derived from the Wuhan-Hu-1 strain (GenBank accession no. MN908947). Stabilization of the spike protein in its prefusion conformation was achieved through proline substitutions at positions K986P and V987P within the S2 hinge region, along with R682S and R685G substitutions at the S1/S2 cleavage site to remove the furin cleavage motif. The replication-incompetent Ad26 vector, with deletions in the E1 and E3 regions, was generated using the AdVac system,^65^ employing a single plasmid approach that incorporates the Ad26 genome along with the transgene expression cassette. A human codon–optimized gene encoding the stabilized spike protein was inserted at the E1 locus of the Ad26 vector genome. The vaccine was produced in the complementing cell line PER.C6 TetR.^66^

The full-length spike protein used for rhesus macaque immunizations (COR200099;^64^) was expressed in Expi293F cells. It is based on the same Wuhan-Hu-1 sequence and was stabilized by the same K986P and V987P proline substitutions and the mutations R682A and R685G. Furthermore, its transmembrane and cytoplasmic domains were replaced by a fibritin foldon domain to promote trimerization, and a C-tag was added to facilitate purification as a soluble protein. Both the adenoviral vectors and protein preparations were screened for bioburden and endotoxin content before administration.

#### LC-MS/MS analysis of purified hamster and macaque IgG

To assess the relative levels of IgG subclasses, total IgG was purified from commercially available Syrian golden hamster serum (ab7483, Abcam, Cambridge, UK) and from a previously studied serum pool of seven healthy rhesus macaques^42^^,^^54^ as previously described,^11^ with minor adjustments. The samples were dried at 45°C as opposed to 60°C. Samples were reduced and alkylated prior to digestion by adding 20 μl 25 mM ammonium bicarbonate (ABC) to each well. Then, sequentially, 5 μl 10 mM DTT (incubated 30 min, 60°C), 5 μl 36 mM IAA (30 min, in dark), 5 μl 42 mM DTT (30 min), were added before overnight digestion using 5 μl 60 ng/μl TPCK trypsin (Promega, cat. no. V5111) (all chemicals were dissolved in 25 mM ABC).

Purified IgG samples were analyzed by RP-LC-ESI-MS/MS and data were processed as previously described^67^ with minor adjustments. Approximately 100 ng of IgG in 0.1% formic acid was analyzed by online C18 nanoHPLC MS/MS with a system consisting of an Ultimate3000nano gradient high-performance liquid chromatography (HPLC) system (Thermo Fisher Scientific, Bremen, Germany), and an Exploris480 mass spectrometer (Thermo Fisher Scientific, Bremen, Germany). Fractions were injected onto a cartridge precolumn (300 μm × 5 mm, C18 PepMap, 5 μm, 100 A, and eluted via a homemade analytical nano-HPLC column (50 cm × 75 μm; Reprosil-Pur C18-AQ 1.9 μm, 120 A; Dr. Maisch, Ammerbuch, Germany). The gradient was run from 2% to 40% solvent B (20/80/0.1 water/acetonitrile/formic acid (FA) v/v) in 50 min. The nano-HPLC column was drawn to a tip of ∼10 μm and acted as the electrospray needle of the MS source. The mass spectrometer was operated in data-dependent MS/MS mode for a cycle time of 3 s, with a HCD collision energies at 30% and recording of the MS2 spectrum in the orbitrap, with a quadrupole isolation width of 1.2 Da. In the master scan (MS1) the resolution was 120,000, the scan range 400-3500, at standard AGC target at maximum fill time of 100 ms. A lock mass correction on the background ion m/z = 445.12003 was used. Precursors were dynamically excluded after *n* = 1 with an exclusion duration of 10 s and with a precursor range of 10 ppm. Charge states 1–7 were included. For MS2 the first mass was set to 110 Da, and the MS2 scan resolution was 30,000 at an AGC target of 100%@maximum fill time of 60 ms. In addition, triggered MS/MS (HexNAc loss (204.087)) was used applying stepped NCE of 20%, 30% and 50% combined to one spectrum in an m/z range 110– 3,500.

For the post-analysis process, databases consisting of the annotated IgG subclasses for hamsters and macaques were generated (sequences as in Table S1). Raw data were converted to peak lists using Proteome Discoverer version 2.5.0.400 (Thermo Fisher Scientific) and submitted to the IgG databases using sequest HT for peptide identification, using the Fixed Value PSM Validator. Sequest searches were with 5 ppm and 0.02 Da deviation for precursor and fragment mass, respectively, with trypsin selected as enzyme and carbamidomethyl as static modification. The abundances of each IgG subclass were divided by total abundance of all subclasses to determine the relative amount of each subclass.

To confirm the identity of sugar structures based on fragmentation spectra, purified IgG from Syrian golden hamster serum (ab7483, Abcam, Cambridge, UK) and from a previously studied serum pool of 7 healthy rhesus macaques,^42^^,^^54^ both not reduced and alkylated, was analyzed by RP-LC-ESI-MS/MS as previously described.^68^ Briefly, the tryptic digests were separated with a Dionex Ultimate 3000 HPLC system (Thermo Fisher Scientific). The system was equipped with an Acclaim PepMap 100 trap column and an Acclaim PepMap RSLC C18 nanocolumn (Thermo Fisher Scientific). The system was coupled to a MaXis quadrupole time-of-flight MS (q-TOF-MS; Bruker Daltonics) equipped with a nanobooster (Bruker Daltonics).

#### Capture, purification, and LC-MS analysis of total and anti-S IgG

Total and anti-S IgG from hamsters and macaques were captured and purified as previously described using our standard protocols.^11^^,^^69^ Briefly, to capture anti-S IgG, Maxisorp plates (Thermo Fisher Scientific) were coated using Spike protein that was produced as described before.^70^ Next, 250 μl Spike protein in PBS-T (1 μg/ml) was added and incubated at 4°C overnight. First, wells were washed with 250 μl PBS supplemented with 0.5% TWEEN 20 (PBS-T). Serum (20 μl) was added to 180 μl PBS-T in the coated plates and incubated for 1 h at 37°C. Wells were washed three times with 250 μl PBS-T, twice with 250 μl PBS, and twice with 250 μl 50 mM ammonium bicarbonate (ABC). Antibodies were eluted with 200 μl 100 mM formic acid. Total IgG was captured using 50 μl Protein G (ProtG) Sepharose beads (GE Healthcare) per well in a 96-well filterplate (Orochem). Beads were washed three times with 200 μl PBS before loading 21 μl sample (20 μl PBS + 1 μl serum) and incubating 1 h while shaking (1000 rpm). Beads were washed three times with 200 μl PBS, three times with ultrapure water, and antibodies were eluted with 200 μl 100 mM formic acid. All eluted antibodies were dried for 2-3 hours at 50°C or 60°C. For both the total and anti-S IgG, digestion was performed using 300 ng of TPCK trypsin in 40 μl 25 mM ABC per well.

Samples were analyzed by LC-MS on a Dionex Ultimate 3000 HPLC system coupled to an Impact HD Q-TOF-MS (Bruker Daltonics) as previously described.^11^ Briefly, 5 μl of Total IgG digest or 5 μl of spike-specific IgG digest was injected and the glycopeptides were separated with solvent A (0.1% TFA in water) and solvent B (95% ACN) with a flow of 0.6 μl/min and the following two-step linear gradient: 0 min: 3% eluent B, 4.5 min: 21.7% eluent B, 5.5 min: 50% eluent B, 8.0 min: 50% eluent B, 9.0 min: 3% eluent B, 11.5 min: 3% eluent B. For the elution range from 3 to 8 min of the LC run data were recorded by the coupled Impact HD quadrupole time-of-flight-MS (q-TOF-MS; Bruker Daltonics) equipped with a nanoBooster (Bruker Daltonics). Ionization was enhanced by applying acetonitrile-doped nebulizing nitrogen gas at 0.2 bar. Profile spectra were recorded in an *m/z* range from 550 to 1800 with a frequency of 1 Hz. The collision energy was 5 eV, the transfer time 110 μs and the pre-pulse storage 21 μs.

#### LC-MS data processing

Raw data analysis was performed in DataAnalysis version 5.0 (Bruker Daltonics). Data were processed as previously described.^11^ Raw files were converted to mzXML files using the MSConvert function of ProteoWizard (version 3.0.8708). The mzXML files were used for the data processing using the in-house developed software LaCyTools (version 1.99).^56^ The alignment of retention times was based on a minimum of four abundant glycoforms for the hamsters and seven for the macaques. For the mass calibration and extraction of glycopeptides, charge states 2-4 were included. For the hamsters, only the first and second isotopes of IgG2 were kept in the analyte list generated by LaCyTools due to the isotopic overlap with IgG1 glycopeptides. Following the data processing using LaCyTools, analytes were curated based the presence in the anti-S samples of both hamsters and macaques. Analytes passed curation when the signal-to-noise ratio was > 9, isotopic pattern quality was < 0.2 (less than 20% deviation from the theoretical isotopic pattern), and the mass error was within 20 ppm in at least 20% of the anti-S samples. For one macaque in group 2, anti-S IgG1 glycopeptides were observed at week 0 (Figure S5). Closer inspection of the LaCyTools output suggested these anti-S signals were artifactual (low isotopic pattern quality and signal-to-noise outside acceptance criteria^56^). Therefore, these anti-S signals were excluded from the analysis. The relative intensities of each glycoform were calculated and the glycosylation traits (fucosylation, bisection, galactosylation, and sialylation) were calculated using the formulas in Table S2.

### Quantification and statistical analysis

Statistical analyses were performed using Rstudio (version 2024.12.1 build 563, Posit Software PBC, Boston, MA) with R (version 4.4.2, R foundation for Statistical Computing, Vienna, Austria). The sample sizes of each group (number of animals) are shown in the figures (Figures 3A and 4A, the statistical tests used, definition of center, and dispersion and precision measures are described in the figure legends. Normality of the data was checked using a Shapiro-Wilk test of normality. Glycosylation traits were compared between timepoints using the Wilcoxon signed-rank test (Figures 3F–3I, 4F–4I, S4, and S6). A Mann-Whitney U test or Welch’s t-test were used to find differences between vaccine types at week 10 (Figure 5). A Spearman’s rank correlation was used to determine the correlations between percentage of glycosylation traits and IgG intensity (Figures S3B–S3E and S5B–S5E). Asterisks indicate the degree of significance with ∗, ∗∗, ∗∗∗, for a *p*-value < 0.05, 0.01, 0.001, respectively. Data were visualized using Rstudio and Illustrator (version 26.3.1, Adobe Inc., San Jose, CA).
