# Supplementary material for: Antigen-specific IgG glycosylation profiles in hamsters and macaques following COVID-19 vaccination
Source: iScience. 2026 Mar 17;29(4):115400. doi: 10.1016/j.isci.2026.115400 (PMC13053761; doi:10.1016/j.isci.2026.115400)
Supplement: Document S1. Figures S1–S6 and Tables S1–S4 [file mmc1.pdf]

iScience, Volume *29*

Supplemental information

Antigen-specific IgG glycosylation profiles in hamsters and macaques following COVID-19 vaccination

Bart Claushuis, Jan Nouta, Wenjun Wang, Carolien A.M. Koeleman, Arnoud H. de Ru, Peter A. van Veelen, Roland Zahn, Ramon Roozendaal, Gestur Vidarsson, and Manfred Wuhrer

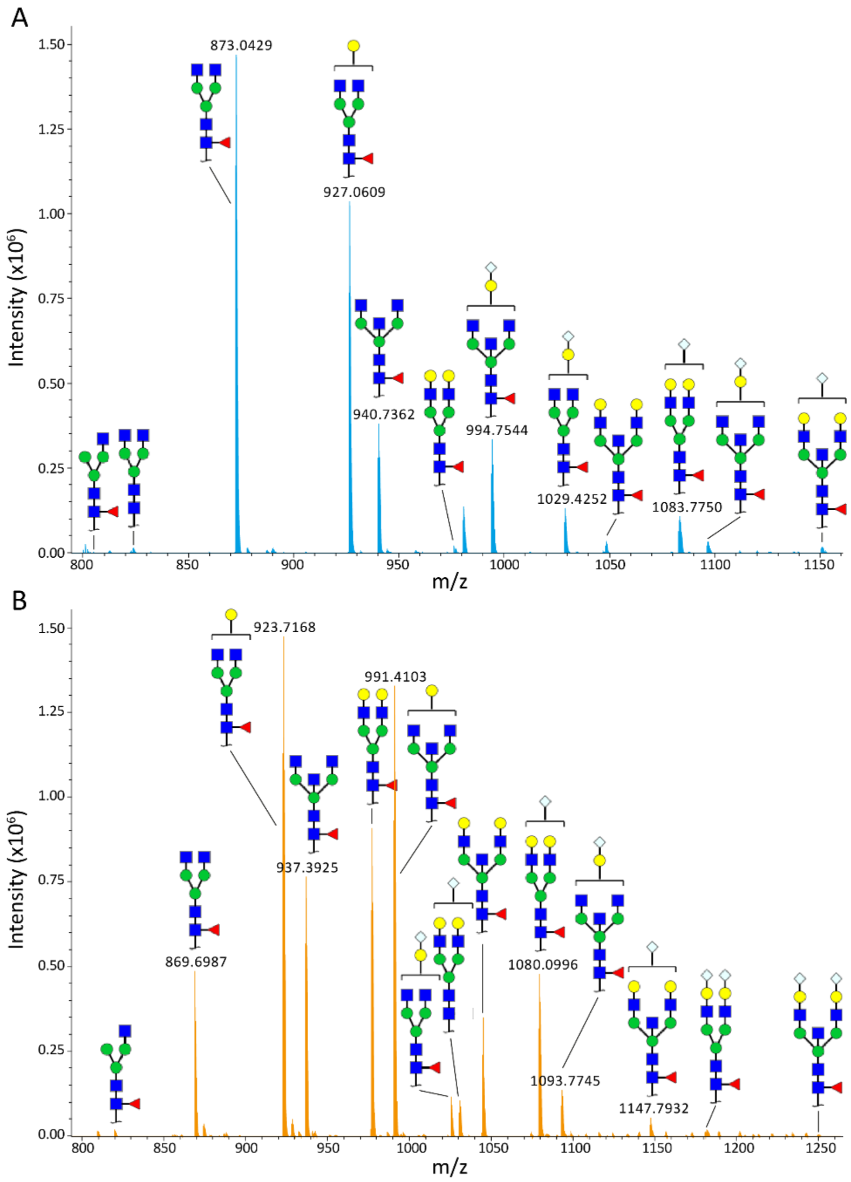

Figure S1. Identification of IgG glycopeptides from serum samples. (**A**) A commercially available serum sample from the Syrian golden hamster and (**B**) a serum pool of 7 healthy rhesus macaques were analyzed using LC-MS and MS spectra were summed. All annotated signals represent 3+ charged species of IgG2 and IgG1 from hamster and macaque, respectively. Glycopeptides were assigned based on accurate mass and are depicted as follows: blue square = N-acetylglucosamine; red triangle = fucose; green circle = mannose; yellow circle = galactose; light-blue diamond = N-glycolylneuraminic acid (sialic acid).

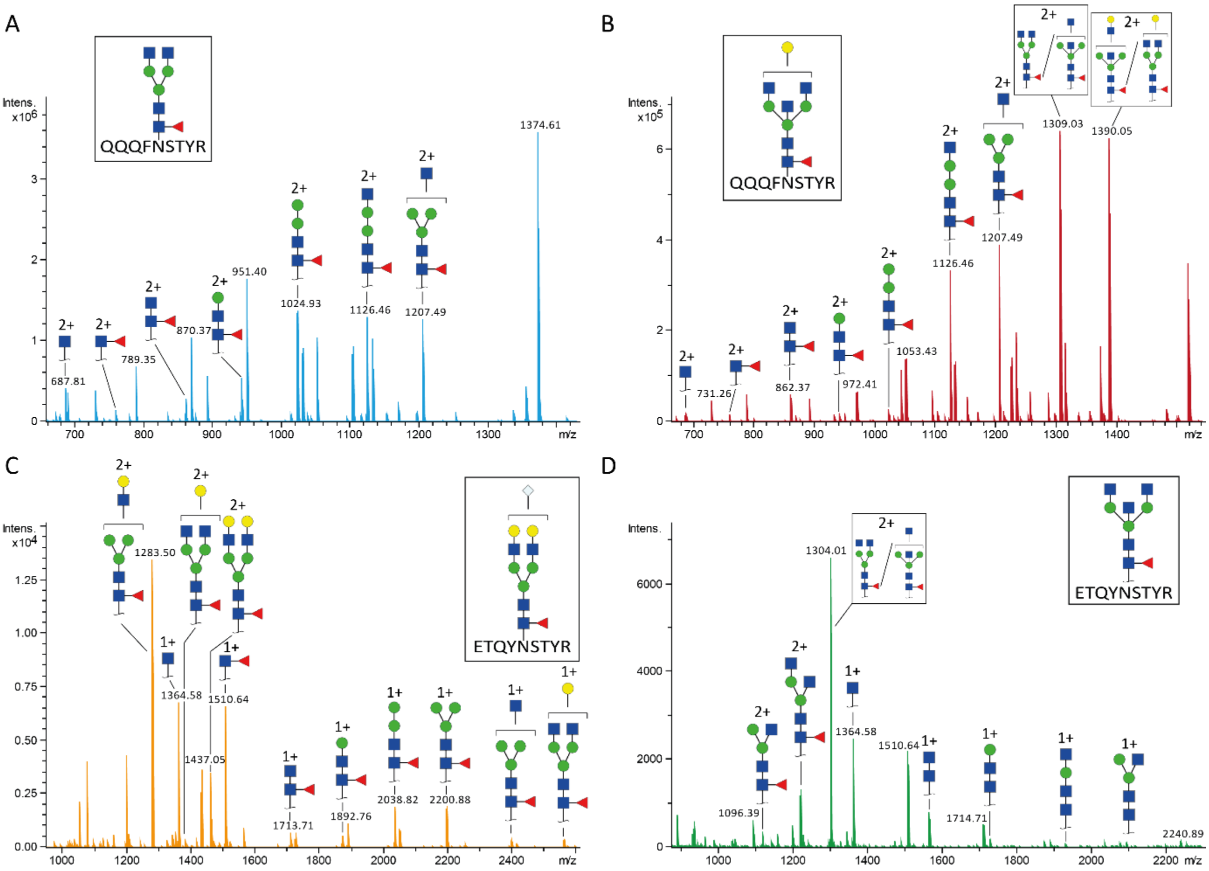

Figure S2. MS/MS spectra of several tryptic Fc glycopeptides. MS/MS spectra for (**A**) hamster IgG2-H3N4F1, (**B**) hamster IgG2-H4N5F1, (**C**) macaque IgG1-H5N4F1G1, and (**D**) Macaque IgG1-H3N5F1. Glycopeptides are depicted as follows: blue square = N-acetylglucosamine; red triangle = fucose; green circle = mannose; yellow circle = galactose; light-blue diamond = N-glycolylneuraminic acid (sialic acid).

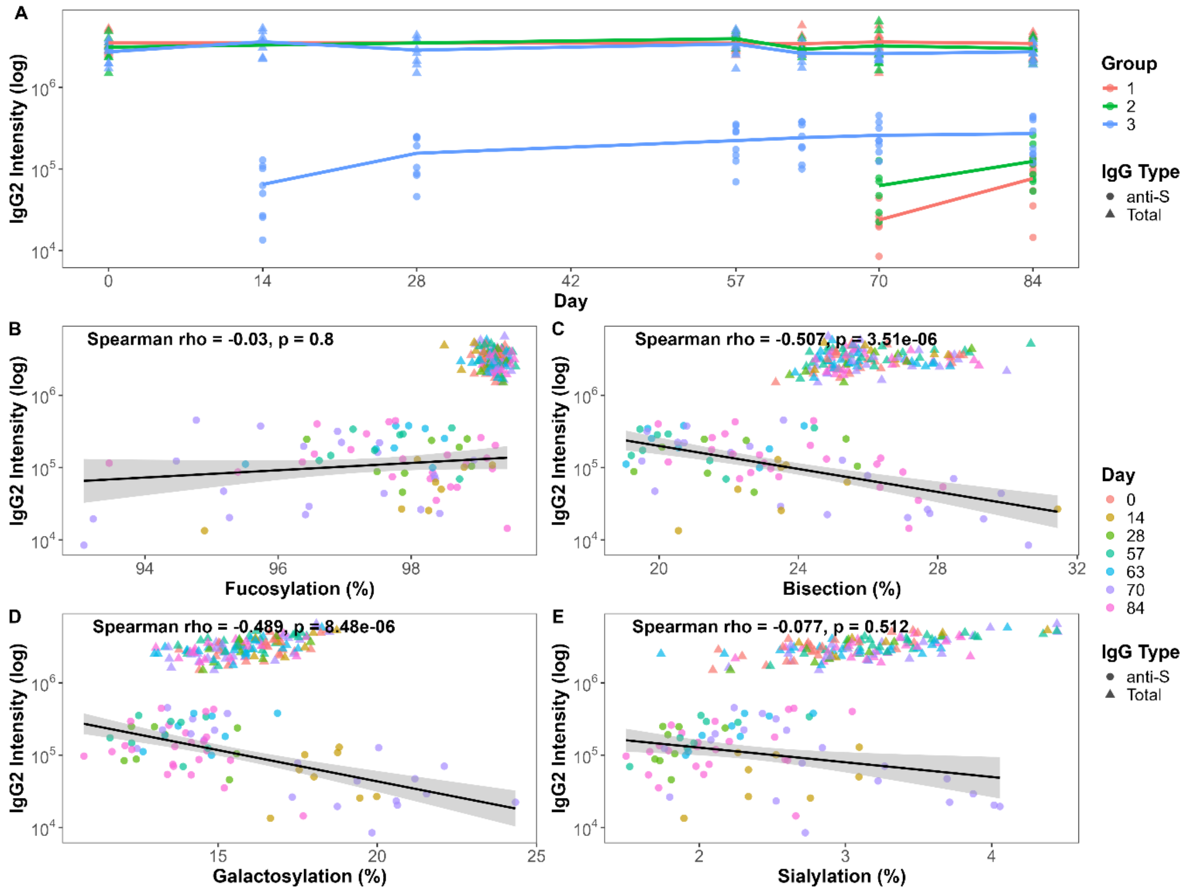

Figure S3. IgG2 intensities and correlations with the glycosylation traits in hamsters. (**A**) The levels of total and anti-S IgG2 over time. The solid lines indicate the mean IgG2 intensity per group. (**B-E**) Correlation between the percentage of the glycosylation traits and IgG2 intensity. The mean anti-S IgG2 and the standard error are depicted by the solid black line and grey ribbon, respectively. The correlation between IgG2 intensities and glycosylation traits was assessed for the anti-S IgG1 using the Spearman's rank correlation coefficient.

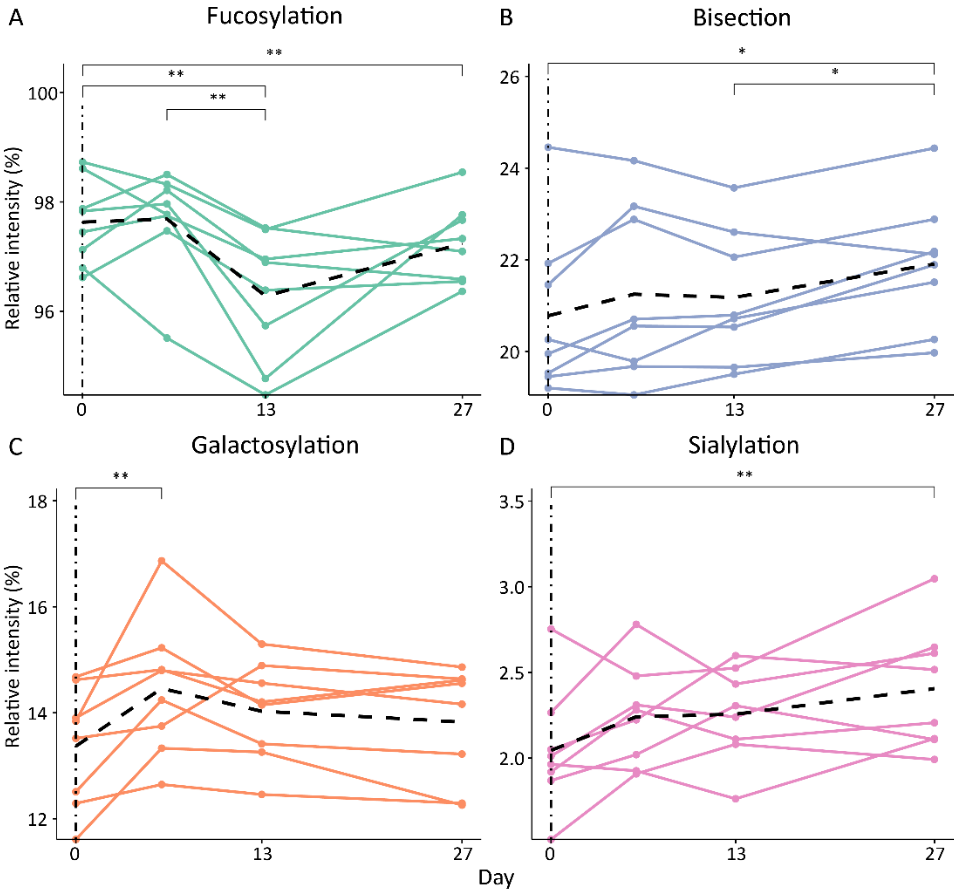

Figure S4. Dynamics of anti-S IgG2 glycosylation after the second dose of Ad26.COV2.S in hamsters from group 3. The vertical dashed lines indicate the day of vaccination, which is considered as day 0. All timepoints were compared to each other using the Wilcoxon signed-rank test. Significance bars are only displayed for statistically significant differences. *$P < 0.05$; **$P < 0.01$; ***$P < 0.001$.

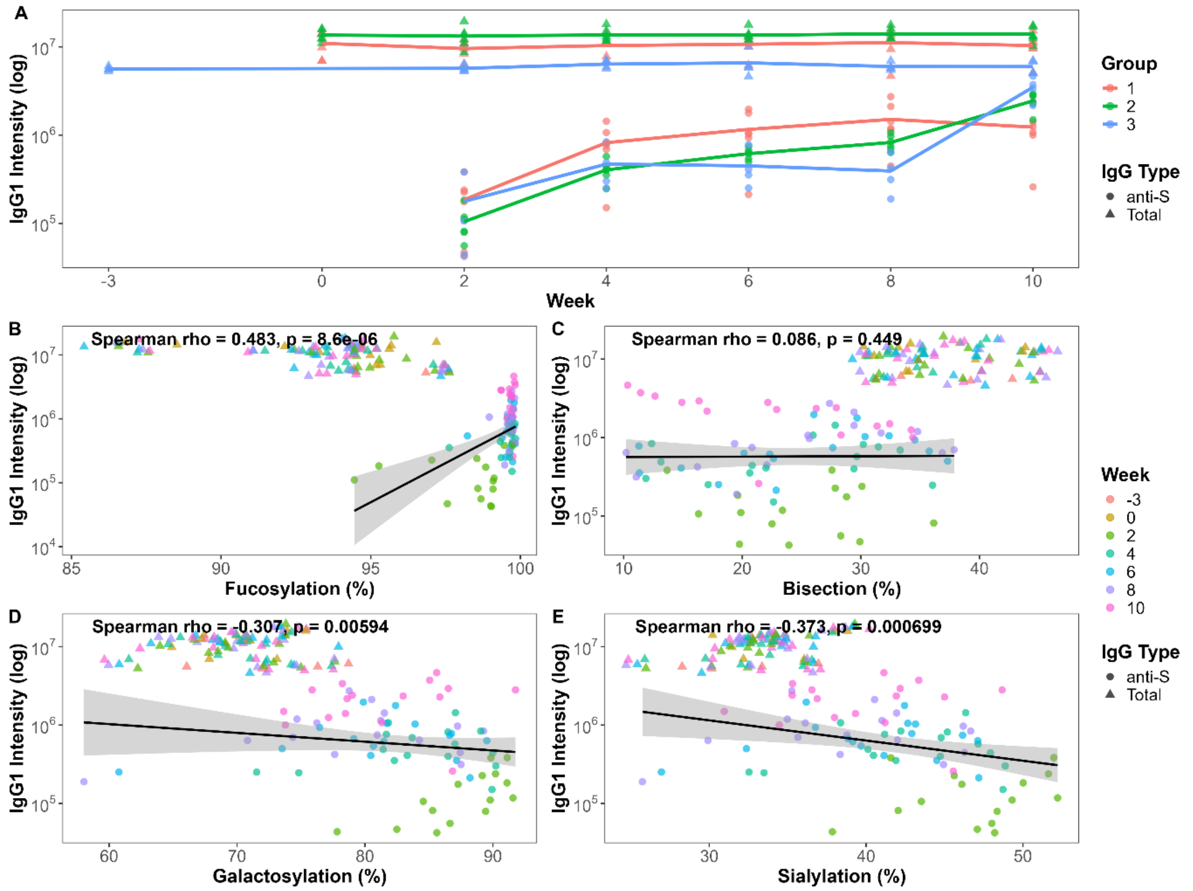

Figure S5. IgG1 intensities and correlations with the glycosylation traits in macaques. (**A**) The levels of total and anti-S IgG2 over time. The solid lines indicate the mean IgG1 intensity per group. (**B-E**) Correlation between the percentage of the glycosylation traits and IgG1 intensity. The mean anti-S IgG1 and the standard error are depicted by the solid black line and grey ribbon, respectively. The correlation between IgG1 intensities and glycosylation traits was assessed for the anti-S IgG1 using the Spearman's rank correlation coefficient.

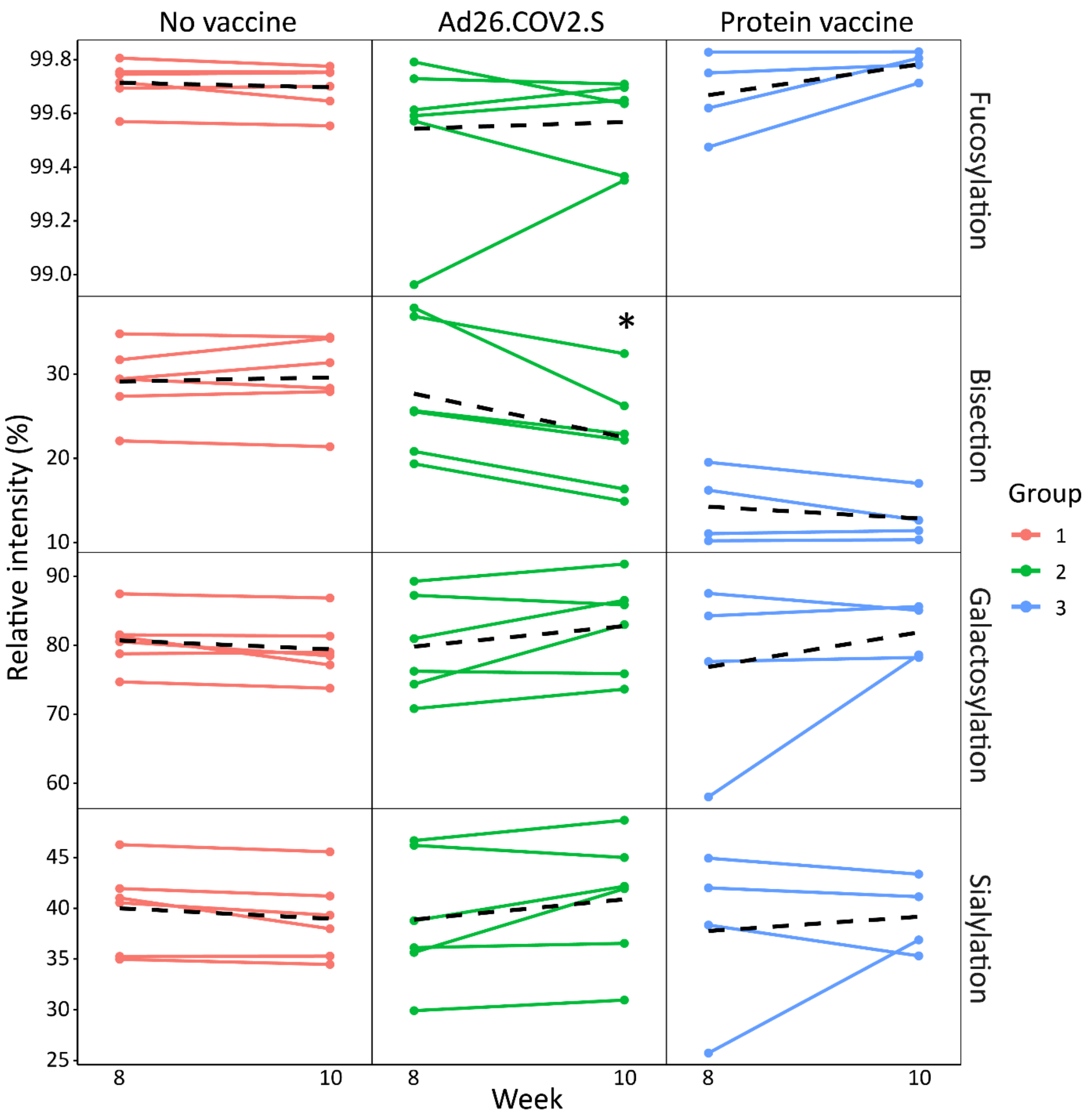

Figure S6. Dynamics of anti-S IgG1 glycosylation after the second dose of Ad26.COV2.S and the protein vaccine in macaques. Black dotted lines connect the mean values at each timepoint. The glycosylation traits at week 8 and 10 were compared using the Wilcoxon signed-rank test. Significance is only displayed for statistically significant differences, which is the case for bisection in group 2. *$P < 0.05$; **$P < 0.01$; ***$P < 0.001$.

Table S1. IgG subclasses in Syrian Golden hamsters and Rhesus macaques

Organism	Accession	Protein name	Sequence
Syrian Golden hamster (*Mesocricetus auratus)*	WEL32134.1	immunoglobulin heavy constant gamma 1, secreted form, partial	LAATTTAPSVYPLAPVLRGTPDSTTVTLGCLVKGYFPEPVTVSWNSGALTSGVHTFPSVLHSGLYSLSSSVTVPSSTWPSQTVTCNVAHPASSTKVDKKIVPGTGDHCKPCVCPGPEVSSVFIFPPKPKDVLTISRSPKVTCVVVDISQDDPEVRFSWFVDGKEVHTAQTQPREEQFNSTYRMVSVLPILHQDWLSGKEFKCKVNSPAFPAPIEKTISKSRGQLQVPQVYAMPPPKEQLTKDEVSLTCMAVGFFPEDIDMEWQRNGEPEKNFRNTPPVLDADETYFLYSKLNVKRKDWESGNSFTCYVVHEALHNHHTEKTLSHRPGK
	WEL32136.1	immunoglobulin heavy constant gamma 2, secreted form, partial	ATTTAPSVYPLAPGGTPDSTTVTLGCLVKGYFPEPVTVSWNSGALTSGVHTFPSVLHSGLYSLSSSVTVPSSTWPSQTVTCNVAHPASSTKVDKKIEPRSCTSLPTLCPKCPAPDLLGGPSVFIFPPNPKDVLTISLTPKVTCVVVDVSEDEPDVQFNWFVNNVEVKTAETQPRQQQFNSTYRVVSSLPIQHQDWLSSKEFKCKVNNKALPSPIEKTISKPRGQARIPQVYTLPPPTEQMTQKVVSLTCMITGFFPADVHVEWEKNGQPEQNYKNTSPVLDTDGSYFMYSKLNVPKSSWEQGNIYVCSVLHEALRNHHTTKAISRSLGN
	WEL32132.1	immunoglobulin heavy constant gamma 3, secreted form, partial	AAGTTAPTVYPLAPACGSTPDSTTVTLGCLVKGYFPEPVTVSWNSGALTSGVHTFPSVLPSGLYSLSSSVTVPSSTWPETITCNVAHLASSTNLNKRIEPRVCSGPQCSCKSFKSPSLVPGNDCHKLSRMGGPVSDPSYLSSLASCIGGPSVFIFPPNPKDVLTISLTPKVTCVVVDVSEDEPDVQFNWFVNNVEVKTAETQPRQQQFNSTYRVVSVLPILHQDWLSGKEFKCKVTNKALPSPVEKTISKPRGQPRVPQVYAMPPPPEQMSKKKVSLTCMITNFFPEDIHVEWERSGELAQDFKNTPPVLDSDGSHFLYSKLTVDTNSWLRGDTFTCTVVHETLRNHYTQKTLSHSPGK
Rhesus macaque (*Macaca mulatta*)	A0AA48P936	IGHG1*03 (Fragment)	STKGPSVFPLAPSSRSTSESTAALGCLVKDYFPEPVTVSWNSGSLTSGVHTFPAVLQSSGLYSLSSVVTVPSSSLGTQTYVCNVNHKPSNTKVDKRVEIKTCGGGSKPPTCPPCPAPELLGGPSVFLFPPKPKDTLMISRTPEVTCVVVDVSQEDPDVKFNWYVNGAEVHHAQTKPRETQYNSTYRVVSVLTVTHQDWLNGKEYTCKVSNKALPAPIQKTISKDKGQPREPQVYTLPPSREELTKNQVSLTCLVKGFYPSDIVVEWESSGQPENTYKTTPPVLDSDGSYFLYSKLTVDKSRWQQGNVFSCSVMHEALHNHYTQKSLSVSPGK
	A0AA48PA68	IGHG2*08 (Fragment)	STKGPSVFPLASCSRSTSQSTAALGCLVKDYFPEPVTVSWNSGALTSGVHTFQAVLQSSGLYSLSSVVTVPSSSLGTQTYVCNVVHEPSNTKVDKTVGLPCRSTCPPCPAELLGGPSVFLFPPKPKDTLMISRTPEVTCVVVDVSQEEPDVKFNWYVDGVEVHNAQTKPREEQFNSTYRVVSVLTVTHQDWLNGKEYTCKVSNKALPAPKQKTVSKTKGQPREPQVYTLPPPREELTKNQVSLTCLVKGFYPSDIVVEWASNGQPENTYKTTPPVLDSDGSYFLYSKLTVDKSRWQQGNTFSCSVMHEALHNHYTQKSLSVSPGK
	A0AA48P8J1	IGHG3*07 (Fragment)	STKGPSVFPLASCSRSTSQSTAALGCLVKDYFPEPVTVSWNSGALTSGVHTFPAVLQSSGLYSLSSVVTVPSSSLGTQTYVCNVVHEPSNTKVDKRVEFTPPCGDTTPPCPPCPAPELLGGPSVFLFPPKPKDTLMISRTPEVTCVVVDVSQEDPEVQFNWYVDGAEVHHAQTKPRERQFNSTYRVVSVLTVTHQDWLNGKEYTCKVSNKGLPAPIEKTISKAKGQPREPQVYILPPPQEELTKNQVSLTCLVTGFYPSDIAVEWESNGQPENTYKTTPPVLDSDGSYFLYSKLIVDKSRWQQGNTFSCSVMHEALHNHYTQKSLSVSPGK
	A0AA48P8J7	IGHG4*05 (Fragment)	STKGPSVFPLASSSRSTSESTAALGCLVKDYFPEPVTVSWNSGALTSGVHTFPAVLQSSGLYSLSSVVTVPSSSLGTQTYVCNVVHEPSNTKVDKRVEFTPPCPACPAPELLGGPSVFLFPPKPKDTLMISRTPEVTCVVVDVSQEDPEVQFNWYVDGVEVHNAQTKPRERQFNSTYRVVSVLTVTHQDWLNGKEYTCKVSNKGLPAPIEKTISKAKGQPREPQVYILPPPQEELTKNQVSLTCLVTGFYPSDIAVEWESNGQPENTYKTTPPVLDSDGSYLLYSKLTVNKSRWQPGNIFTCSVMHEALHNHYTQKSLSVSPGK

Table S2. Description and calculation of IgG1 glycosylation traits. H: hexose, N: N-acetylhexosamine, F: fucose, G: N-glycolylneuraminic acid

Derived trait	Description	Formula hamsters	Formula macaques
**Fucosylation**	*N*-glycans carrying a core fucose	H3N4F1/(H3N4+H3N4F1)	H5N4F1G1 / (H5N4G1 + H5N4F1G1)
**Bisection**	*N*-glycans carrying a bisecting *N*-acetylglucosamine	(H3N5F1+H4N5F1) / Sum of all glycopeptides	(H3N5F1 + H4N5F1 + H5N5F1 + H4N5F1G1 + H5N5F1G1 + H5N5F1G2) / Sum of all glycopeptides
**Galactosylation**	*N*-glycans carrying one or two galactoses	(H4N4F1+H4N5F1+H4N4F1G1) * 0.5 + (H5N4F1+H5N4F1G1) / Sum of all glycopeptides	(H4N4F1 + H4N5F1 + H4N4F1G1 + H4N5F1G1) * 0.5 + (H5N4F1 + H5N4G1 + H5N5F1 + H5N4F1G1 + H5N5F1G1 + H5N4F1G2 + H5N5F1G2) / Sum of all glycopeptides
**Sialylation**	*N*-glycans carrying one or two *N*-glycolylneuraminic acids (sialic acid)	(H4N4F1G1+H5N4F1G1) * 0.5 / Sum of all glycopeptides	(H4N4F1G1 + H5N4G1 + H5N4F1G1 + H4N5F1G1 + H5N5F1G1) * 0.5 + (H5N4F1G2 + H5N5F1G2) / Sum of all glycopeptides

Table S3. Hamster IgG2 glycopeptides included in the analyte list

Glycan composition	Alternative nomenclature	[M+2H]$^{2+}$	[M+3H]$^{3+}$	Proposed structure
**H3N3F1**	G0F-N	1207.0054	805.0060	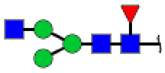
**H3N4**	G0	1235.5161	824.0132	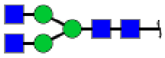
**H3N4F1**	G0F	1308.5451	872.6991	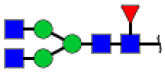
**H4N4F1**	G1F	1389.5715	926.7168	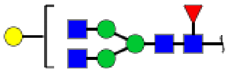
**H3N5F1**	G0FN	1410.0848	940.3923	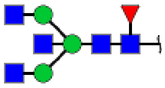
**H5N4F1**	G2F	1470.5979	980.7344	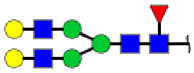
**H4N5F1**	G1FN	1491.1112	994.4099	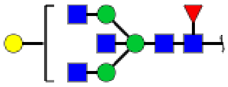
**H4N4F1G1**	G1FS1	1543.1167	1029.0802	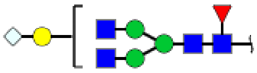
**H5N4F1G1**	G2FS1	1624.1431	1083.0978	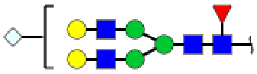
**H4N5F1G1**	G1FNS1	1644.6563	1096.7733	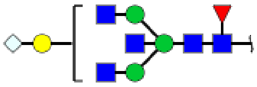

Table S4. Macaque IgG1 glycopeptides included in the analyte list

Glycan composition	Alternative nomenclature	$[M+2H]^{2+}$	$[M+3H]^{3+}$	$[M+4H]^{4+}$	Proposed structure
**H3N4F1**	G0F	1303.5291	869.3552	652.2682	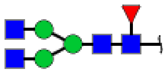
**H4N4F1**	G1F	1384.5555	923.3728	692.7814	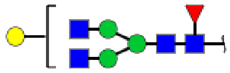
**H3N5F1**	G0FN	1405.06	937.0483	703.0380	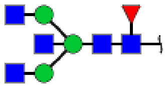
**H5N4F1**	G2F	1465.5820	977.3904	733.2946	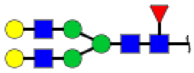
**H4N5F1**	G1FN	1486.0952	991.0659	743.5513	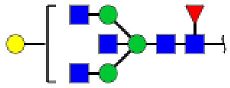
**H4N4F1G1**	G1FS1	1538.1007	1025.7362	769.5540	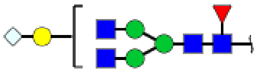
**H5N4G1**	G2S1	1546.0982	1031.0679	773.5527	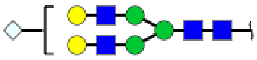
**H5N5F1**	G2FN	1567.1216	1045.0835	784.0645	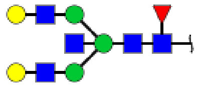
**H5N4F1G1**	G2FS1	1619.1271	1079.7538	810.0672	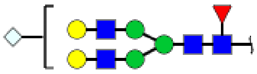
**H4N5F1G1**	G1FNS1	1639.6404	1093.4294	820.3238	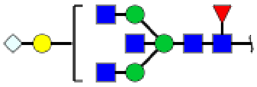
**H5N5F1G1**	G2FNS1	1720.6668	1147.4470	860.8370	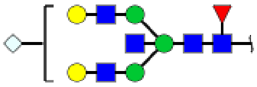
**H5N4F1G2**	G2FS2	1772.6723	1182.1173	886.8398	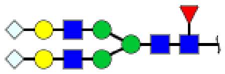
**H5N5F1G2**	G2FNS2	1874.2120	1249.8104	937.6096	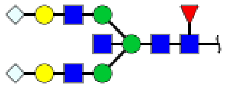
